# Herpes Zoster—Ophthalmicus

**Published:** 1873-07

**Authors:** A. W. Calhoun

**Affiliations:** Vienna, Austria


### HERPES ZOSTER, OPHTHALMICUS.

BY A. W. CALHOUN, M.D., VIENNA, AUSTRIA.

A very peculiar and painful affection of the skin is here and there met with under the name of herpes zoster, which makes its appearance upon any and every portion of the body, but when taking its seat in the region of the eye, and producing certain more or less important and serious effects hereafter to be mentioned, upon the bulbus itself, receives the additional name ophthalmicus. To clearly comprehend this important disease, a few words in explanation of the usual accompanying skin affection, as it precedes or rapidly follows the eruption upon the conjunctiva and cornea, will be necessary.

When occurring upon the frontal or nasal region, the beginning and progress is the same as when situated upon the back, its favorite seat, or any other part of the skin. Along the course of one or more of the terminal branches of the ophthalmic division of the trigeminus, or fifth pair of nerves, as a rule the supra-orbitalis, and frequently extending far up into the scalp as high as the vertex of the head, can be traced numberless smaller and larger vesicles, filled in the beginning with watery contents. The vesicles mark out the direction of the nerves, belng arrayed to some extent regularly, from the exit of the nerve out of the foramen, to its termination. It not unfrequently happens that the naso-ciliary branch is at the same time implicated, the vesicles extending down on the side of the nose to its end, and on the adjacent parts. When this is the case the eye is said to become more rapidly and more immediately affected, from the direct connection of the bulbus with the naso-ciliary nerve through the ciliary ganglion.

In the part in which the disease is about to make its appearance the patient has a stinging, burning sensation, the skin assumes a red, swollen appearance, and is exceedingly sensitive to the touch. This sensitiveness increases in a short time to a violent neuralgia in those parts supplied by the affected terminal branches of the trigeminus, and continues for a greater or less period, two or three days, till the eruption takes place upon the skin, when the neuralgic pain either entirely ceases or greatly diminishes. The eruption consists of single groups of vesicles which remain separate and distinct as groups, or several running together unite in a confluent mass. The vesicles, gradually bursting and drying, form a scabby surface, the groups succeeding upon other portions of the course of the nerve passing through the same change. After the scabs fall away, very frequently a deep and often painful cicatrix remains behind, which, by its contraction and pulling upon the delicate nervous fibers, is a constant cause of neuralgia. These cicatrices are quite characteristic, appearing as if the fingernail, or some similarly formed instrument, had been used in digging out the skin and underlying subcutaneous tissue, and remaining deepened after healing. Such characteristic cicatrices are of life-long duration, and, as above remarked, always the source of neuralgic pain. By old persons these results of herpes zoster are more marked than in younger individuals, the pain being of greater duration and more intense.

In such instances the affection of the eye follows a few days after the eruption upon the skin in the vicinity, but occasionally is it the case that the first precedes the latter in appearance. The conjunctiva becomes much inflamed, and, after a short interval, the inflammation extends into the cornea, producing small ulcers either upon its edge or toward the center, the first position being the most frequent, which, in the most favorable case, leave behind, after healing, an opacity corresponding in extent to the size of the original ulcer. But the termination of the disease upon the eye is not always so favorable, iritis, with its manifold subsequent evils, being a not uncommon follower of the corneal affection. The ulcers sometimes destroy the cornea to such a depth that a rupture takes place, the thinned corneal tissue not being able to resist the pressure of the aqueous humor from behind, and, as a consequence of this giving away at the bottom of the ulcer, a prolapsus iridis follows, which, when of large size and uniting with the edges of the ulcer and healing in this condition, is often the starting point of a staphiloma. A similar and very interesting case is now under treatment in the eye clinic, in the person of an old man near sixty years of age. The skin affection began in accordance with the above description, the eye disease following in one or two days after the eruption. Upon his entering the clinic, ulceration had already begun upon the cornea, and, in the face of the most careful and energetic treatment, rupture resulted upon two of the places occupied by the ulcers, with subsequent prolapse of the iris, iritis, etc., etc. The acute symptoms of the disease in this case having passed away, the treatment is now mainly directed against the formation of a staphiloma, to prevent which, as may be supposed, is no easy task, since the destruction of the cornea is extensive and the size of the prolapsus large.

The disease occurs mostly upon one side of the face and in one eye only, but the possibility of its occurrence upon both sides and in both eyes, either at the same time or after a short interval, can be readily imagined.

The treatment consists in alleviating the suffering of the patient, produced by the neuralgia, by means of sedative applications, such as an ointment in which is mixed a sufficient amount of opium and belladonna plaster, etc., etc., spread upon the inflamed parts of the face. Where such treatment is insufficient to reduce the pain, and this is but too often the case, it is best at once to make use of the hypodermic injection of morphine, which seldom fails, if only for a short time, to put the individual at rest.

The eye itself must be treated according to the damage it has suffered or with which it is threatened. As the ulceration of the cornea follows in quick succession the appearance of the disease upon the face, we usually see the patient after the keratitis is more or less farther advanced. At this stage the best treatment is the frequent use of atropine dropped into the eye, and absolute rest and quiet for the organ. If the ulcers are deep and extensive, it is also, in addition, necessary to keep the eye under a moderately firm bandage, which prevents the lids from moving back and forth over the ulcerated parts, and thus hindering a more rapid cure. Thus, by means of the bandage, one of the chief conditions for the most satisfactory healing is gained, viz: the requisite quiet for the eye. Other symptoms which may, in unfavorable cases, later appear, such as rupture of the cornea, and prolapsus iridis, etc., must be managed as their extent and degree of violence may demand, but when the disease is in time taken in hand, most cases can be brought safely through under the above simple treatment.

